# Efficacy of Magnesium Sulfate as an Adjuvant to Bupivacaine in Transversus Abdominis Plane Block for Abdominal Hysterectomy Surgeries

**DOI:** 10.7759/cureus.37156

**Published:** 2023-04-05

**Authors:** Komalea Priya Balakrishna, Nirmala Devi Kagalkar, Anusha Suntan

**Affiliations:** 1 Anesthesiology, Shri B M Patil Medical College and Hospital, Vijayapura, IND; 2 Anesthesiology, Shri B M Patil Medical College and Hospital, Bijapur Lingayat District Educational (BLDE) University, Vijayapura, IND; 3 Anesthesiology, Shri B M Patil Medical College and Hospital, Bijapur Lingayat District Educational (BLDE) Univeristy, Vijayapura, IND

**Keywords:** magnesium sulfate, total abdominal hysterectomy, enhanced recovery procedure, visual analogue scale, tap block

## Abstract

Background

Major abdominal surgeries are often accompanied by excruciating pain, which, if not adequately managed, can reduce patient comfort and satisfaction, delay mobilization, compromise respiratory and cardiac functioning, and increase healthcare costs. The transversus abdominis plane (TAP) block is an efficient and safe complement to multimodal postoperative analgesia for abdominal surgery. This study evaluates the efficacy of combining magnesium sulfate (MgSO_4_) with bupivacaine for TAP block in patients posted for total abdominal hysterectomy (TAH).

Methodology

Seventy female patients between the ages of 35 and 60 who were scheduled to have a TAH under spinal anesthesia were divided randomly into two groups of 35 each: group Bupivacaine (B) and group Bupivacaine-Magnesium sulfate (BM). Group B received 18 milliliters (mL) of bupivacaine 0.25 percentage (%) 45 milligrams (mg) with 2 mL normal saline (NS) whereas group BM received 18 mL of bupivacaine 0.25% (45 mg) with 1.5 mL of 10% weight/volume (w/v) MgSO_4_ (150 mg) and 0.5 mL NS in the ultrasonography-guided (USG) bilateral TAP block performed after the end of surgery. Groups were compared for the postoperative visual analog scale (VAS) scores, the time required for first rescue analgesia, the number of analgesic rescues at various time intervals, patient satisfaction score, and any side effects.

Results

Postoperative VAS scores at 4, 6, 12 and 24th hour (hr) (p < 0.05) in group BM were lower compared to group B. Time required for rescue analgesia was significantly prolonged in group BM (882.94 ± 70.22 minutes) compared to group B (459 ± 100.53 minutes) with minimal usage of rescue analgesia (p < 0.05) up to 12 hr. In group BM, the patient satisfaction score was higher (p = 0.001).

Conclusion

In addition to a considerable reduction in post operative VAS scores and overall use of rescue analgesia, the addition of magnesium to bupivacaine significantly prolongs the TAP block and increases the initial postoperative period of bearable pain.

## Introduction

Most of the patients scheduled for surgery endure emotional stress due to their fear of postoperative pain [1]. Total abdominal hysterectomy (TAH) is accompanied by an extensive inflammatory response resulting in postoperative discomfort and misery [2,3]. Such patients require appropriate analgesic treatment to reduce morbidity and complications by blunting autonomic, somatic, and endocrine reflexes [4]. As a result, postoperative analgesia following TAH calls for a multimodal approach.

Numerous studies have demonstrated that when enhanced recovery procedures (ERPs) are used, hospital length of stay, time to return to normal function, postoperative ileus duration, thromboembolic complications, morbidity, and all of these factors are all reduced [5]. In order to achieve the best pain treatment, many ERPs use a multimodal approach, decreasing the use of opioids as the primary analgesic in favor of neuraxial and regional anesthetic techniques [6]. One of the regional techniques routinely used is the transversus abdominis plane (TAP) block. Its widespread use in abdominal surgeries is due to its technical simplicity and trustworthy analgesia [7,8]. This block implies administering local anesthetic between the internal oblique muscle and transversus abdominis muscle, which is marked by the lumbar Petit triangle. The primary drawback of single-shot regional blocks is their short duration of action when administered with local anesthetic alone. Several adjuvants, including opioids, alpha 2 agonists, N-methyl-D-aspartate (NMDA) receptor antagonists, and other drugs, have been utilized to extend the duration of blocks [9,10]. Opioids are by far the most often used adjuvants, but they accompany a host of unpleasant side effects, such as respiratory depression, drowsiness, nausea, and vomiting. In some studies, using alpha 2 agonists such as Dexmedetomidine and Clonidine has been related to drowsiness and bradycardia [11].

Magnesium is a calcium blocker and an NMDA receptor antagonist. Some studies have shown that magnesium reduces sensitivity to central or peripheral pain stimulation by inhibiting calcium influx and excitability of NMDA receptors [12]. Despite possessing analgesic qualities, magnesium's use as a local anesthetic adjuvant in peripheral nerve blocks has not been widely investigated. Hence this study was carried out to assess the efficacy of magnesium sulfate (MgSO_4_) as an adjuvant to bupivacaine in ultrasonography (USG)-guided TAP block in patients scheduled for TAH.

## Materials and methods

This was a prospective double-blinded randomized comparative study conducted in Shri BM Patil Medical College, Hospital and Research Centre, Vijayapura, India from January 2021 to April 2022, after obtaining approval from the institutional ethics committee IEC/NO-09/2021. Seventy female patients aged between 35 and 60 years, of American Society of Anesthesiologists (ASA) I & II status, posted for TAH under spinal anesthesia were enlisted in the study. The patient's refusal, any bleeding problem or patients taking anticoagulants, local infection at the block's site, any cardio-respiratory conditions, convulsions, and neurological deficits were among the exclusion criteria. The patients were randomly allocated into two equal groups of 35 each by computer-generated randomization table. Group B (Bupivacaine) received a USG-guided bilateral TAP block with 18 milliliters (mL) of bupivacaine 0.25 percentage (%) 45 milligram (mg) with 2 mL normal saline (NS) and group BM (Bupivacaine - Magnesium sulfate) received USG guided bilateral TAP block with 18 mL of bupivacaine 0.25% (45 mg) with 1.5 mL of 10% weight/volume (w/v) MgSO4 (150 mg) and 0.5 mL NS.

In the pre-operative visit, a thorough preanesthetic check-up was done and the study protocol and procedures were explained to the patients. All patients received an explanation of the Visual Analogue Scale (VAS) scoring. All patients who took part in the research provided their written informed consent. Prior to the operation, all patients were kept fasting orally for six hours. On arrival at the operation theater 18-gauge, intravenous (IV) cannula was secured and Ringer’s lactate solution was infused with 15 milliliters/kilogram (mL/kg) body weight. A premedication of 0.08 mg/kg body weight of Midazolam and 50 mg of Ranitidine was administered intravenously. Standard monitoring devices were attached like a pulse oximeter, sphygmomanometer cuff, and electrocardiography lead, and baseline readings were noted.

Under all aseptic conditions, patients undergoing TAH were given spinal anesthesia in the left lateral position using a 25-gauge Quincke spinal needle at L3-L4 interspace. After confirming the free flow of cerebrospinal fluid, 15 mg of 0.5% hyperbaric bupivacaine was injected. After confirmation of adequate level, surgery was started. Following the procedure, a bilateral USG-guided TAP block was carried out under strict aseptic precautions, and 20 mL of study solution was injected into each side. After draping the abdominal region between the 12th rib and anterior superior iliac crest with the umbilicus in the center, the external oblique muscle, internal oblique muscle, transversus abdominis muscle, and their fascia were located beneath the skin and subcutaneous tissue using a linear high-frequency probe 6-13 Megahertz of the SonoSite Micromax machine. A 23-gauge spinal needle was advanced using the in-plane technique at the anterior axillary line, and the precise position of the needle tip between the internal oblique and transverse abdominis muscle was visualized. To expand the plane, 2 mL of normal saline was administered. Following the affirmation of a hypoechoic region on the USG image, the study solutions were injected as per group allocation. 

An investigator blinded to the group assignment evaluated patients in the post-anesthesia care facility for pain, heart rate, blood pressure, and any side effects such as nausea, vomiting, and hematoma at time 0 (time of completion of TAP block), 1, 2, 4, 6, 12, and 24 hours. Postoperatively, both groups of patients received an injection of Paracetamol 1 gram (gm) intravenously every 8th hourly as a part of multimodal analgesia. Whenever the VAS ≥4 or else on patient demand, injection of Diclofenac 75 milligram intramuscular (i.m) was given as rescue analgesia. If still the patient's VAS score was ≥4, Tramadol 2 mg/kg i.v was administered.

The primary outcome of the study was the postoperative VAS score between the two groups. The secondary outcome includes the time required for the first rescue analgesia, the total doses of rescue analgesic consumption, patient satisfaction score, and any side effects like postoperative nausea, vomiting, myositis, and hematoma. VAS comprises a 10-centimeter (cm) line with 1 cm markings on each end, on which the patient makes a mark to indicate the level of pain that she is feeling. Mark 0 for no pain and mark 10 for the worst possible pain. The patient's numbers were read as units of pain intensity measurement. On a four-point measure, patients were asked to describe the severity of their nausea and vomiting: None (0) Mild (1) Moderate (2) Severe (3) Very severe (4). Patients were asked to evaluate their satisfaction with pain management on a five-point scale 24 hours after surgery: Highly dissatisfied (1) Dissatisfied (2) Neither dissatisfied nor satisfied (3) Satisfied (4) Highly satisfied (5).

Statistical analysis

The sample size was calculated using the VAS score as the main variable. The anticipated Mean ± SD (standard deviation) of the VAS score at the 6th hour in group BM is 2.4 ± 1.33 and in the group, B is 4.53 ± 2.62. To obtain a power of 90% and a level of significance of 5%, a minimum sample size of 35 per group (i.e., a total sample size of 70, assuming equal group sizes) is needed. All the data were analyzed using the statistical package for the social sciences (SPSS) software v.23.0. All characteristics were descriptively summarized. Summary statistics of mean, SD was used for continuous variables. For categorical data, the Freeman-Halton Fisher exact test and the chi-square test were used to assess the significance of group differences. The difference between the means of the analysis factors between the two independent groups was determined using an unpaired t-test. The findings were deemed clinically statistically significant if the p-value was less than 0.05.

## Results

**Figure 1:**
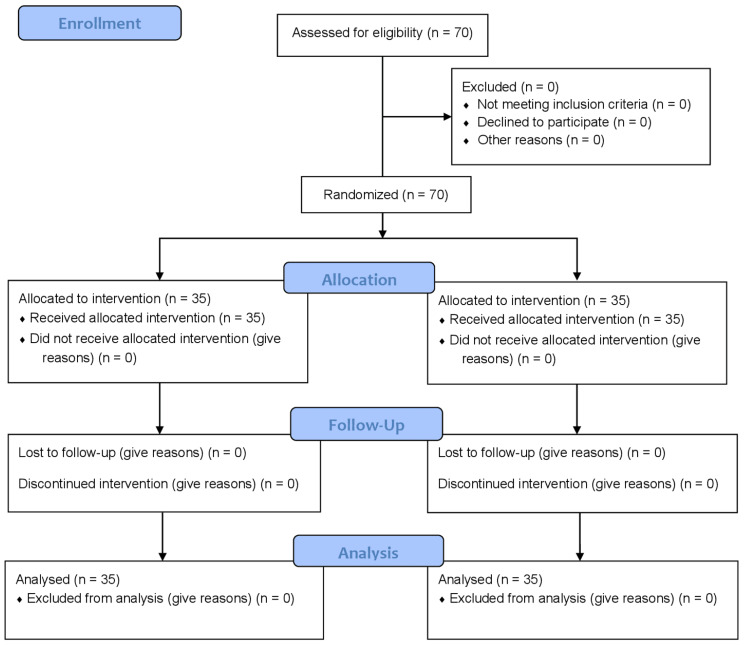
CONSORT flow diagram. Consolidated Standards of Reporting Trials (consort), number (n)

 After screening and randomization, 70 patients were included in the study, 35 in each group. The Consolidated Standards of Reporting Trials (CONSORT) flow diagram is shown in Figure 1.

Regarding demographic parameters in Table 1 like age, weight, height, body mass index, ASA status and duration of surgery were similar in both the groups and statistically insignificant. 

**Table 1:** Demographic data of patients in both the groups. Group B (Bupivacaine), Group BM (Bupivacaine-Magnesium sulfate), standard deviation (SD), kilograms (kg), centimeters (cm), Body mass index (BMI), kilogram per meter square (kg/m^2^), minutes (mins)

DEMOGRAPHIC PARAMETERS	GROUP B	GROUP BM	P-VALUE
	MEAN±SD	MEAN±SD	
AGE (Years)	42.25 ± 5.30	42.62 ± 5.926	0.845
WEIGHT (kg)	57.88 ± 7.30	56.37 ± 7.62	0.196
HEIGHT (cm)	161.25 ± 6.63	160.54 ± 7.74	0.663
BMI (kg/m^2^)	22.38 ± 3.28	21.95 ± 2.77	0.557
DURATION OF SURGERY (mins)	113.42 ± 14.48	113.57 ± 14.27	1.00

VAS score was found to be less in group BM compared to group B for 24 hours postoperative period, and it was statistically insignificant at 0,1,2 hours and significant at 4,6,12, and 24 hours. Group B had a higher VAS in the sixth hour (4.51 ± 0.95) and group BM had a higher VAS in the 12th hour (3.13 ± 1.55) as shown in Table 2.

**Table 2:** Comparison of mean VAS scores at different time intervals between group B and group BM. Visual Analogue score (VAS), Standard deviation (SD), Hour (HR), Bupivacaine (B), BM (Bupivacaine-Magnesium sulfate)

VAS	GROUP B	GROUP BM	P-VALUE
	MEAN±SD	MEAN ± SD	
IMMEDIATE POSTOPERATIVE	0.28 ± 0.32	0.11 ± 0.45	0.076
1 HR	0.54 ± 0.49	0.37 ± 0.50	0.127
2 HR	1.17 ± 0.85	1.02 ± 0.29	0.427
4 HR	2.40 ± 1.31	1.74 ± 0.70	0.001
6 HR	4.51 ± 0.95	2.48 ± 0.78	0.001
12 HR	3.87 ± 1.14	3.13 ± 1.55	0.026
24 HR	3.19 ± 0.93	2.14 ± 0.34	0.001

**Figure 2:**
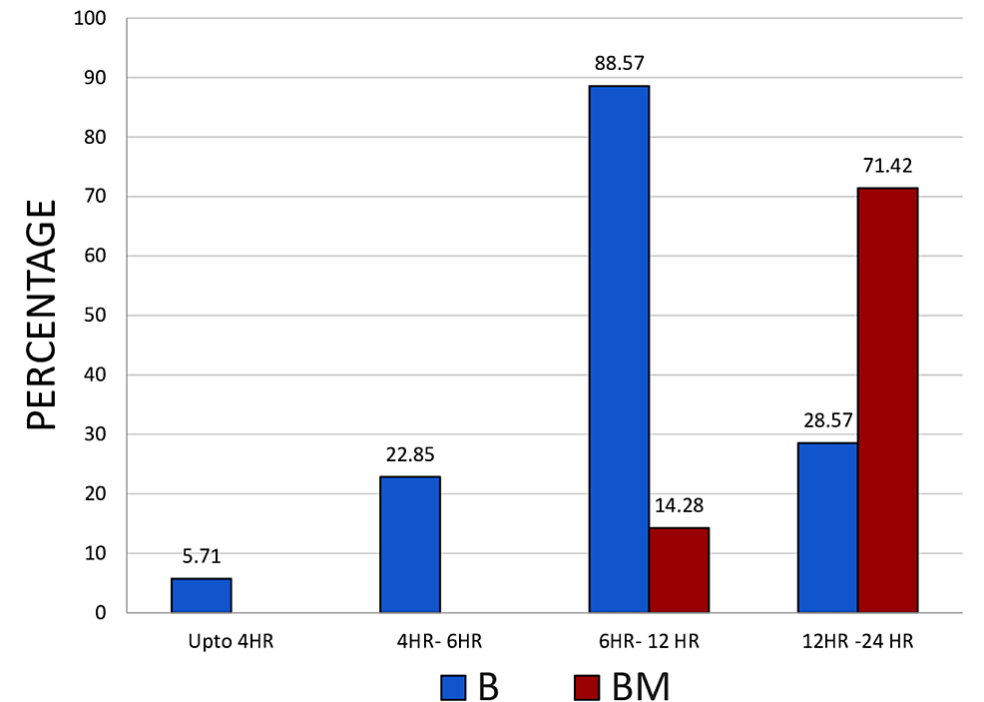
Comparison of Analgesic Requirements at Different Time Intervals Between Group B and Group BM. Bupivacaine (B), Bupivacaine Magnesium Sulphate (BM), Hour (HR)

 In first 4 hr, there were two demands for rescue analgesic in group B (5.71%) and none in group BM. Between 4 and 6 hr, eight patients in group B (22.85%) demanded rescue analgesia but none in group BM. Between 6 and 12 hr, 31 patients in group B (88.57%) and five in group BM (14.28%) demanded rescue analgesia. Between 12 and 24 hr, 10 patients in group B (28.57%), and 25 patients in group BM (71.42%) demanded rescue analgesia (Figure 2). In first 12 hr, group B required more rescue analgesics than group BM. Patients of BM needed rescue analgesia after 12 hr. None of the patients from either group needed second rescue analgesia in the form of i.v Tramadol.

Table 3 shows the time for first rescue analgesia was significantly increased in group BM (882.94 ±70.22 mins) compared to group B (459 ± 100.53 mins), which was clinically and statistically highly significant. Comparison of mean post‑operative nausea and vomiting (PONV) score in both groups, noticed more in group B compared to group BM, (1.16 ± 0.39 vs. 1.06 ± 0.26), which was statistically insignificant as shown in Table 3. Patient satisfaction score was better in group BM (4.22 ± 0.73) compared to group B (3.37± 0.77), which was statistically significant as shown in Table 3.

**Table 3:** Study outcome. Bupivacaine (B), Bupivacaine-Magnesium sulfate (BM), Minutes (mins)

PARAMETER	GROUP B	GROUP BM	P-VALUE
	MEAN ± SD	MEAN ± SD	
Time to first rescue analgesia (mins)	459 ± 100.53	882.94 ± 70.22	<0.001
Postoperative nausea vomiting score	1.16 ± 0.39	1.06 ± 0.26	0.477
Short assessment of patient satisfaction score	3.37 ± 0.77	4.22 ± 0.73	<0.001

## Discussion

This randomized clinical trial demonstrated that the bilateral TAP block with magnesium sulfate as an adjuvant to bupivacaine provides effective postoperative analgesia for patients undergoing TAH. It lowered the severity of pain and the need for rescue analgesia after surgery. All blocks were performed USG-guided, ensuring visualization of the entire needle, and repeated aspiration to avoid complications. In this study, demographic data and duration of surgery of both groups were statistically insignificant. The main finding in this study was that group B had higher VAS scores than group BM. Up to 2 hr, VAS scores in both groups were statistically insignificant, which may be due to residual effects of sub arachnoid block. This study shows VAS scores were significantly less 6th, 12th, and 24th hr after the block in group BM compared to group B. It shows that adding magnesium sulfate to bupivacaine substantially reduces VAS pain scores up to 24 hours (p = 0.001). Rana et al. [13] conducted the same study in total abdominal hysterectomy patients and reported that the addition of 150 mg of magnesium sulfate reduced the post-operative VAS score in 4, 6, and 12 hr (P < 0.05). Abd-Elsalam et al. [14] conducted a prospective, randomized, double-blind study on 60 women undergoing total abdominal hysterectomy. Over the first 24 hours after surgery, the MgSO4 group had a substantially lower postoperative VAS scores than the bupivacaine group. Bondok et al. [15] noticed that intra-articular administration of MgSO4 (500mg) in arthroscopic knee surgery reduced postoperative VAS scores. Additionally, it delays the first rescue analgesic request and reduces the demand for further analgesic drugs. During the first 8 hr after surgery, the intra-articular magnesium group had lower VAS scores than the intra-articular saline group. In this study, group B needed the most rescue analgesia between the sixth and 12th hr, whereas group BM required more rescue analgesia after 12 hours of post-surgery. It suggests a brief interval of pain relief and an increased need for rescue analgesic with plain bupivacaine. Therefore, magnesium typically decreased the need for analgesics on the first postoperative day.

 The main mechanism could be magnesium's voltage-dependent antagonistic effects on NMDA receptors, which regulate calcium ion influx and prevent central sensitization of peripheral nociceptive stimulation, resulting in less intense pain after acute inflammation [16]. Another proposed theory is that magnesium enhances excitatory neurotransmitter release at the synaptic junction, which may enhance the effects of local anesthetics [17,18]. The surface charge hypothesis also contributes to the understanding of magnesium sulfate's analgesic effects on peripheral neurons. Magnesium ions have been shown to increase the firing threshold in both myelinated and unmyelinated neurons. It has been proposed that divalent cations decrease the fixed negative surface charge of the outer neuronal membrane, raising the transmembrane potential and resulting in hyperpolarization [19]. When a fiber is hyperpolarized, it is more difficult for it to achieve the threshold level, resulting in a conduction block. Akutagawa et al. [20] demonstrated that modulating the exogenous magnesium content surrounding a nerve bundle improved nerve blockade caused by local anesthetics. Elshahaly et al. [19] reported that magnesium sulfate as an adjuvant to TAP block in patients posted for unilateral open inguinal hernia repair had less requirement of rescue analgesia postoperatively.

 The magnesium-dose employed in this investigation was based on data from the Gunduz A et al. study [21]. He concluded that adding 150 mg of magnesium to prilocaine increases the duration of the axillary plexus block compared to 100mg of magnesium without causing systemic effects or neurotoxicity. Similar to this, Goyal et al. [22] reported that MgSO_4_ was used as an adjuvant in the axillary block and that it was given in doses of 200 mg and 100 mg. The length of analgesic relief in the MgSO_4_ groups was longer than in the control groups (p<0.05). Despite the fact that pain relief with 200 mg MgSO_4_ lasted longer (193.83 ± 294.11 mins) than with 100 mg MgSO_4_ (85.33 ± 144.38 mins) and morphine use was lower in both research groups (16.97 ± 6.61 mg, 24.17 ± 7.52 mg) than in the control group (28.97 ± 11.65 mg) (p<0.05). Umalkar et al. [23] employed 75 mg of MgSO_4_ to bupivacaine as an adjuvant in TAH cases and found that it did not extend the duration of postoperative analgesia, which might be due to inadequate dosage of magnesium sulfate. In this study, the usage of MgSO_4_ 150 mg in group BM provided good postoperative analgesia, with increased time to first rescue analgesia compared to group B. In this study also, the PONV score was similar between the two groups. A statistically significant difference in terms of mean patient satisfaction score was found higher in group BM. No injury to the viscera or hematoma was observed in this study.

Limitations

An area of limitations in this study was that serum Magnesium levels were not evaluated, and the study group was less in number. To generalize the results large group studies are required.

## Conclusions

In conclusion, MgSO_4_ can be considered an effective adjuvant to bupivacaine in TAP block in patients posted for TAH under spinal anesthesia. It resulted in good analgesia as determined by reduced VAS scores, prolonged time for first rescue analgesia, and less demand for rescue doses with better patient satisfaction scores. Neither group experienced local anesthetic toxicity, hematomas, or excessive tissue trauma at the injection site. As a result, we advocate for the use of MgSO_4_, which is more easily accessible in most operating rooms and less expensive. This research suggests that MgSO_4_ is a helpful adjuvant to long-acting local anesthetics for TAP block.
